# Hydroxylated Alkyl and Phenyl Phosphonium Ionic Liquids Exhibit Enhanced Antibacterial and Anti-Biofilm Activity

**DOI:** 10.3390/antibiotics15070655

**Published:** 2026-07-01

**Authors:** Oscar Forero-Doria, Rosío Rodríguez-Azúa, Maria Parot-Cabrera, Verónica Olate-Olave, Christina Mitsi, Ricardo I. Castro, Matías Monroy-Cárdenas, Whitney Venturini, Ramiro Araya-Maturana, Luis Guzmán

**Affiliations:** 1 Departamento de Bioquímica Clínica e Inmunohematología, Facultad de Ciencias de la Salud, Universidad de Talca, Talca 3460000, Chile; oforero@utalca.cl (O.F.-D.); javiparot@gmail.com (M.P.-C.); volate@utalca.cl (V.O.-O.); 2 Oral and Maxillofacial Histopathology Laboratory, Department of Stomatology, Faculty of Dentistry, Universidad de Talca, Talca 3460000, Chile; rorodriguez@utalca.cl; 3 Doctorado en Ciencias Biomédicas, Facultad de Ciencias de la Salud, Universidad de Talca, Talca 3460000, Chile; 4 Magister en Ciencias Biomédicas, Facultad de Ciencias de la Salud, Universidad de Talca, Talca 3460000, Chile; 5 Center for Bioinformatics and Integrative Biology, Facultad de Ciencias de la Vida, Universidad Andrés Bello, Santiago 8370146, Chile; christina.mitsi@usach.cl; 6 Multidisciplinary Agroindustry Research Laboratory, Carrera de Ingeniería en Construcción, Instituto de Ciencias Aplicadas, Universidad Autónoma de Chile, Talca 3467987, Chile; ricardo.castro@uautonoma.cl; 7 Laboratorio de Síntesis y Reactividad de Compuestos Orgánicos, Departamento de Química, Facultad de Ciencias Exactas, Universidad Andrés Bello, Santiago 8370146, Chile; maty.monroy@gmail.com; 8 Departamento de Medicina Traslacional, Facultad de Medicina, Universidad Católica del Maule, Talca 3460000, Chile; wventurini@ucm.cl; 9 MIBI (Interdisciplinary Group on Mitochondrial Targeting and Bioenergetics), Universidad de Talca, Talca 3460000, Chile; raraya@utalca.cl; 10 Instituto de Química de Recursos Naturales, Universidad de Talca, Talca 3460000, Chile

**Keywords:** phosphonium ionic liquids, antibacterial activity, anti-biofilm activity

## Abstract

The rapid emergence of antimicrobial resistance has intensified the search for alternative antimicrobial scaffolds that target both planktonic bacteria and biofilm-associated infections. In this study, a series of hydroxylated phosphonium ionic liquids derived from triphenylphosphonium (TPP^+^) and trihexylphosphonium (THP^+^) cations bearing C3, C6, C7, and C10 ω-hydroxyalkyl chains were synthesized and evaluated for their antibacterial and anti-biofilm activities. Antibacterial activity was determined using broth microdilution assays against *Staphylococcus aureus* and *Escherichia coli*, while anti-biofilm activity was assessed by disrupting preformed biofilms using a 96-pin microtiter plate system and crystal violet staining. The results showed that antibacterial activity was strongly influenced by the amphiphilic balance of the compounds, particularly the alkyl chain length and the nature of the phosphonium core. Derivatives bearing C6OH–C10OH chains exhibited the highest antibacterial activity, whereas short-chain analogs displayed markedly reduced potency. THP derivatives were notably more active against *E. coli* bacteria, consistent with their higher hydrophobicity and activity consistent with membrane interaction. In addition, THP derivatives demonstrated greater biofilm disruption, achieving up to ~90% biomass removal in *E. coli* biofilms, with C6OH–C7OH derivatives showing the most favorable activity profile. Hemolysis assays indicated low erythrocyte toxicity at concentrations close to antibacterial MIC values, indicating a favorable selectivity window. Overall, these findings highlight phosphonium ionic liquids as promising antimicrobial agents with activity consistent with membrane interaction and provide structure–activity insights for the rational design of new antibacterial and anti-biofilm compounds.

## 1. Introduction

The treatment of infectious diseases, which still pose a major global health burden, is increasingly undermined by the rapid emergence of multidrug-resistant (MDR) bacteria and the stagnation in the discovery and development of novel antibiotics [1,2]. Under these circumstances, the optimization of existing therapeutic strategies is imperative. A key aspect of this effort is the rational use of antibiotics, administered at appropriate doses and durations to ensure therapeutic efficacy while minimizing the development of antimicrobial resistance (AMR) [3]. However, since the onset of the COVID-19 pandemic, antimicrobial stewardship has been severely challenged by substantial increases in antibiotic prescribing. Reports indicate that more than 71% of hospitalized COVID-19 patients in China received antibiotics despite a bacterial co-infection rate of only 1% [4], while in other cohorts, antibiotic use reached up to 95% even though secondary infections were confirmed in merely 15% of cases [5,6].

In this context, quaternary heteronium salts (QHSs), including quaternary ammonium and phosphonium compounds, have emerged as promising chemical scaffolds for alternative antimicrobial agents that address AMR. Concurrently, the COVID-19 pandemic has driven the extensive use of disinfectants aimed at limiting the spread of SARS-CoV-2 and other opportunistic pathogens, particularly formulations containing quaternary ammonium compounds (QACs). Notably, QAC-based products account for nearly 47% of the disinfectants listed by the U.S. Environmental Protection Agency as effective against SARS-CoV-2 [7]. Nevertheless, the massive and prolonged use of QACs has led to the emergence of bacterial tolerance and resistance, first genetically documented in the 1980s. Subsequent studies have demonstrated that reduced susceptibility to QACs is primarily associated with biofilm formation, membrane compositional changes, and the overexpression of efflux systems encoded by *qac* genes [8,9].

In parallel, several quaternary phosphonium salts (QPSs), which share structural and functional similarities with quaternary ammonium salts (QASs), have been developed during the mid-20th century and reported as effective antibacterial agents [10]. Renewed interest in their biological activity has been stimulated by the widespread adoption of phosphonium-based ionic liquids (PILs) in science and technology. Compared to nitrogen-containing ionic liquids, PILs often exhibit advantages such as faster synthetic kinetics and enhanced thermal stability [11]. Notably, long-chain tetraalkylphosphonium salts have demonstrated broad-spectrum antimicrobial activity along with lower cytotoxicity relative to their ammonium analogs [12,13]. In addition, their biological activity has been reported to be less dependent on alkyl chain length. Other phosphonium-based systems, such as tributyl(2-hydroxyethyl) phosphonium docusate, have shown significant antibacterial and anti-biofilm activity against antibiotic-resistant bacterial strains [14]. In this context, although alkyl chain length is often considered a key determinant of antibacterial activity, other structural features play a critical role. In particular, the combination of cationic charge and overall hydrophobicity governs the interaction with bacterial membranes, promoting electrostatic attraction followed by insertion into the lipid bilayer. Previous studies, including those from our group, have shown that substituents on the aromatic moiety and the nature of the cationic core can significantly modulate membrane interactions and antibacterial efficacy, sometimes leading to a non-linear relationship with alkyl chain length [15]. Moreover, it has been reported that once a threshold level of amphiphilicity is reached, further increases in alkyl chain length do not necessarily enhance antibacterial activity, suggesting that membrane saturation effects may become the limiting factor [16]. These observations highlight that antibacterial performance arises from a balance of structural features rather than a single parameter.

Among the most extensively studied phosphonium salts are those based on triphenylphosphonium (TPP^+^) cations that consist of a central phosphorus atom bonded to three phenyl rings, yielding a rigid aromatic framework with partial delocalization of the positive charge across the π systems [17]. This electronic delocalization contributes to enhanced chemical stability and moderate lipophilicity, features that have facilitated their broad exploration in biological applications. Beyond their wide applications in the cancer field [18], phosphonium salts have also attracted interest in other areas of medicinal chemistry [19], including recently reported antiplatelet [20,21] and antifouling properties [12]. Also, it is known for its antibacterial properties [22,23], among other functionalities [24,25]. In contrast, trihexylphosphonium cations comprise a phosphorus center substituted with three long aliphatic hexyl chains, resulting in a flexible and sterically bulky molecular architecture. The extended hydrocarbon chains markedly increase hydrophobicity and favor strong interactions with lipid bilayers, a property that is often correlated with membrane-disruptive antimicrobial mechanisms [26,27,28].

Taken together, these observations highlight that the antimicrobial performance of quaternary phosphonium salts is governed by subtle yet critical structural parameters, including the electronic nature of the phosphonium core and the balance between aromaticity, hydrophobicity, and steric flexibility imparted by the substituent groups. While increasing evidence supports their antibacterial potential, a systematic understanding of how these molecular features translate into membrane interactions, antibacterial potency, and cytotoxicity remains incomplete. In this context, this work aims to elucidate how the nature of the phosphonium core (aromatic versus aliphatic) and the modulation of alkyl chain length influence antibacterial efficacy, structure–activity relationships, and membrane-associated mechanisms of action. By assessing these parameters, the study seeks to provide preliminary structure–activity relationship insights for the rational design of phosphonium ionic liquids with improved antibacterial performance and controlled cytotoxicity.

## 2. Materials and Methods

### 2.1. Synthesis of ω-Hydroxyalkyltriphenylphosphonium Bromides

The previously reported methodology [20] for obtaining phosphonium salts was modified by using a conventionally heated, sealed-vessel reactor instead of reflux heating, thereby significantly diminishing the reaction time (from 24 h to 30 min). Thus, a 10 mL process vial equipped with a magnetic stir bar, containing a mixture of 500 mg of the respective bromoalkyl alcohol and 1.5 equivalents of triphenylphosphine in 3 mL of toluene, was heated at 160 °C for 30 min in a Monowave 50 synthesis reactor (Anton Paar GmbH; Graz, Austria). The supernatant was discarded, and the product was washed with ethyl acetate until all remaining reactants were removed. The following products were obtained: (3-Hydroxypropyl)triphenylphosphonium bromide [29] (93% yield); (6-hydroxyhexyl)triphenylphosphonium bromide [30] (79% yield); (7-hydroxyheptyl)triphenylphosphonium bromide [30] (93% yield); and (10-hydroxydecyl)triphenylphosphonium bromide [31] (77% yield).

### 2.2. Synthesis of ω-Hydroxyalkyltrihexylphosphonium Bromides

The synthesis was carried out in a sealed-vessel reactor. Thus, a 10 mL process vial equipped with a magnetic stir bar, containing a mixture of 500 mg of the respective bromoalkyl alcohol and 1.2 equivalents of trihexylphosphine in 5 mL of acetonitrile, was heated at 130 °C for 30 min in a Monowave 50 synthesis reactor (Anton Paar GmbH; Graz, Austria). The supernatant was discarded, and the product was washed with diethyl ether and hexane until all remaining reactants were removed (3 × 5 mL each).

(3-Hydroxypropyl)trihexylphosphonium bromide (**5a**) 90% yield. ^1^H NMR (400 MHz, DMSO-*d*_6_) δ 3.46 (t, *J* = 5.8 Hz, 2H, CH_2_-OH), 3.40–3.60 (br s, 1H, OH, overlapped), 2.29–2.13 (m, 8H, 4x^+^P-CH_2_-), 1.62 (m, 2H, -CH_2_-CH_2_-OH), 1.47 (m, 6H, 3x^+^P-CH_2_-CH_2_), 1.37 (m, 6H, 3x^+^P-CH_2_-CH_2_-CH_2_), 1.33–1.20 (m, 12H, 3x^+^P-CH_2_-CH_2_-CH_2-_CH_2_-CH_2_), 0.87 (t, *J* = 6.5 Hz, 9H, 3xCH_3_ of hexyl chain); ^13^C NMR (101 MHz, DMSO) δ 60.95, 60.80, 30.88, 30.30, 30.15, 24.64, 24.60, 22.29, 21.05, 21.01, 18.35, 17.88, 15.48, 15.00, 14.33.

(6-Hydroxypropyl)trihexylphosphonium bromide (**5b**) 85% yield. ^1^H NMR (400 MHz, DMSO-d_6_) δ 3.48 (br s, 1H, OH), 3.39 (t, J = 6.3 Hz, 2H, CH_2_-OH), 2.28–2.10 (m, 8H, 3x^+^P-CH_2_-, -CH_2_-CH_2_-OH), 1.48 (m, 8H, 3x^+^P-CH_2_-CH_2_, -CH_2_-CH_2_-CH_2_-OH), 1.38 (m, 11H, 3x^+^P-CH_2_-CH_2_-CH_2_, -CH_2_-CH_2_-CH_2_-CH_2_-CH_2_-OH), 1.30 (m, 12H, 3x^+^P-CH_2_-CH_2_-CH_2-_CH_2_-CH_2_), 0.88 (t, J = 6.7 Hz, 9H, 3xCH_3_ of hexyl chain); ^13^C NMR (101 MHz, DMSO) δ 61.00, 32.62, 30.86, 30.54, 30.39, 30.28, 30.13, 25.23, 22.28, 21.15, 21.11, 21.04, 21.00, 18.24, 17.77, 14.33.

(7-Hydroxypropyl)trihexylphosphonium bromide (**5c**) 72% yield. ^1^H NMR (400 MHz, DMSO-d_6_) δ 3.62 (s, 1H, OH), 3.43–3.27 (m, 2H, CH_2_-OH), 2.20 (m, 8H, 3x^+^P-CH_2_-,-CH_2_-CH_2_-OH), 1.54–1.43 (m, 8H, 3x^+^P-CH_2_-CH_2__,_ -CH_2_-CH_2_-CH_2_-OH), 1.43–1.33 (m, 10H, internal 5x-CH_2_-), 1.34–1.20 (m, 16H, internal 8x-CH_2_- of alkyl chain), 0.88 (t, J = 6.1 Hz, 9H, 3xCH_3_ of hexyl chain); ^13^C NMR (101 MHz, DMSO) δ 61.09, 32.90, 30.86, 30.29, 30.14, 22.28, 21.04, 21.00, 18.24, 17.77, 14.33.

(10-Hydroxypropyl)trihexylphosphonium bromide (**5d**) 63% yield. ^1^H NMR (400 MHz, DMSO-d_6_) δ 3.38 (t, J = 6.5 Hz, 2H, CH_2_-OH), 3.3–3.5 (br s, 1H, OH overlapped), 2.28–2.08 (m, 8H, 3x^+^P-CH_2_-,-CH_2_-CH_2_-OH), 1.48 (m, 8H, 3x^+^P-CH_2_-CH_2__,_ -CH_2_-CH_2_-CH_2_-OH), 1.39 (m, 10H, internal 5x-CH_2_-), 1.34–1.17 (m, 22H, internal 11x-CH_2_- of alkyl chain), 0.88 (t, J = 6.7 Hz, 9H, 3xCH_3_ of hexyl chain); ^13^C NMR (101 MHz, DMSO) δ 61.16, 33.01, 30.86, 30.28, 30.13, 29.50, 29.42, 29.14, 28.61, 25.99, 22.27, 21.02, 20.98, 18.22, 17.75, 14.33.

The ^1^H and ^13^C-NMR spectra for compounds (**5a**–**d**) are available in the Supporting Information (Figures S1–S8).

### 2.3. ATR-FTIR Spectral Acquisition and Preprocessing

Sample spectra were recorded using a PerkinElmer Spectrum Two spectrometer (PerkinElmer, Waltham, MA, USA). A small quantity of sample was applied onto the diamond crystal of the Attenuated Total Reflectance (ATR) accessory, ensuring complete and uniform sample–crystal contact. Between measurements, the ATR crystal was thoroughly cleaned with isopropanol to prevent contamination. Spectral data were collected in the mid-infrared region (4000–525 cm^−1^) using air as background, and instrument parameters included 40 scans per sample at a 4 cm^−1^ resolution. Prior to sample measurements, a background spectrum was recorded to account for the instruments and environmental effects. A 30 min warm-up period preceded spectral acquisition to ensure detector stability.

Sample preprocessing was performed using Omnic 8.2 (Thermo Fisher Scientific Inc., Waltham, MA, USA). The spectra were smoothed using the Savitsky–Golay algorithm (95-point moving second-degree polynomial) in the automatic smoothing option. Subsequently, the spectra were further processed using automatic baseline correction and scale normalization.

### 2.4. Thermal Analysis

Thermogravimetric (TG) analysis of the different ionic liquid samples was conducted using a Discovery STD-600 thermogravimetric analyzer (TA Instruments Inc, New Castle, DE, USA). For each analysis, approximately 10 mg of ionic liquid was placed in a platinum (Pt) crucible. The sample was heated from 50 to 600 °C at a constant rate of 10 °C min^−1^, with nitrogen as a reactive gas at a flow rate of 50 mL min^−1^. Furthermore, the electronic balance was protected with 50 mL min^−1^ of N_2_.

### 2.5. Antibacterial Activity

#### 2.5.1. Bacterial Strains and Culture Conditions

*Escherichia coli* ATCC 25922 and *Staphylococcus aureus* ATCC 29213 were used in this study. The strains were maintained on Mueller–Hinton (MH) agar and subcultured 18–24 h prior to each experiment. Bacterial suspensions were adjusted to a 0.5 McFarland standard (1 × 10^8^ CFU/mL) and subsequently diluted to obtain a final inoculum of 5 × 10^5^ CFU/mL.

#### 2.5.2. Preparation of Phosphonium Ionic Liquid Solution

**Scheme 1:**
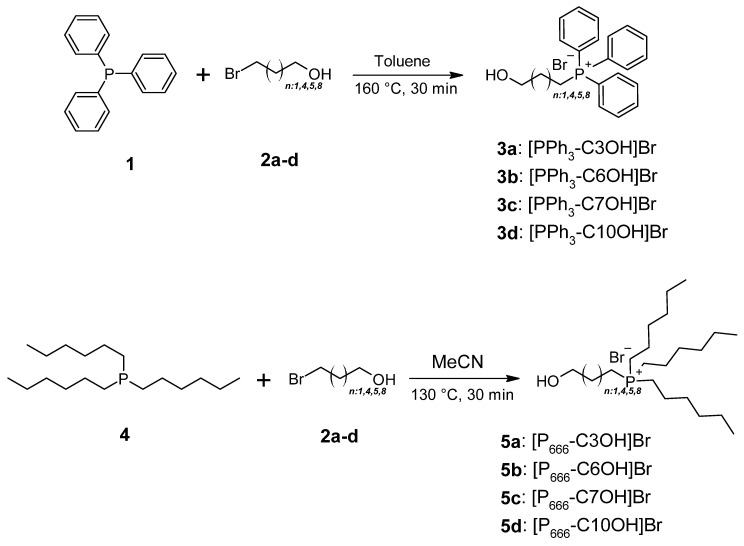
Synthesis of triphenyl- and trihexylphosphonium ionic liquids bearing ω-hydroxyl-terminated alkyl chains of different lengths.

The antimicrobial activity of ionic liquids derived from triphenylphosphonium (TPP^+^) and trihexylphosphonium (THP^+^) cations was evaluated, including the ionic liquids [PPh_3_-C3OH]Br, [PPh_3_-C6OH]Br, [PPh_3_-C7OH]Br, [PPh_3_-C10OH]Br, [P_666_-C3OH]Br, [P_666_-C6OH]Br, [P_666_-C7OH]Br, and [P_666_-C10OH]Br (see Scheme 1).

Each compound was solubilized in 0.9% physiological saline supplemented with 0.5–1% (*v*/*v*) DMSO, according to its solubility profile. Fresh stock solutions were prepared at a concentration of 2 mM prior to each experiment.

#### 2.5.3. Minimum Inhibitory Concentration (MIC) Determination

The minimum inhibitory concentration (MIC) was determined using the broth microdilution method in 96-well microtiter plates, following standard guidelines [32], with methodological considerations based on Wiegand et al. [33] and Kowalska-Krochmal and Dudek-Wicher [34]. Given the presence of ω-hydroxyl groups in the phosphonium ionic liquids, minor adaptations were incorporated based on the colorimetric microdilution approach described by Eloff et al. [35]. Each well contained a final volume of 210 μL, consisting of 100 μL of Mueller–Hinton broth and 100 μL of compound solutions. They were prepared by two-fold serial dilutions in Mueller–Hinton broth, covering a final concentration range from 1 mM to 0.00049 mM (1000–0.49 µM), followed by the addition of bacterial inoculum adjusted to 5 × 10^5^ CFU/mL. Growth controls (bacteria without compound), sterility controls (medium only), and compound sterility controls were included in all experiments. In addition, vehicle controls containing the corresponding DMSO concentrations were included in all assays. The highest final DMSO concentration was 0.5% (*v*/*v*) at the maximum compound concentration tested (1 mM), while lower concentrations contained proportionally lower DMSO levels as a result of the two-fold serial dilution procedure. Plates were incubated at 37 °C for 18–24 h under aerobic conditions.

Following incubation, bacterial growth was first assessed visually based on turbidity relative to the growth and sterility controls. To facilitate endpoint determination and assess metabolic activity, 2,3,5-triphenyl-tetrazolium chloride (TTC) was added as a colorimetric viability indicator. The MIC was defined as the lowest concentration of the compound that showed no visible bacterial growth, which was further supported by the absence of TTC-derived red coloration, indicating a lack of detectable metabolically active cells under the assay conditions. All MIC determinations were performed in at least three independent experiments to ensure reproducibility of the observed endpoints.

#### 2.5.4. Biofilm Formation and Maturation Using a 96-pin Microtiter Plate Lid System

The evaluation of biofilm disruptive activity was performed using a 96-pin microtiter plate lid system, following the method described by Tsukatani et al. [36], with minor adaptations. The same bacterial strains, culture conditions, and ω-hydroxylated phosphonium ionic liquids used in the MIC assays were employed in this experiment, all grown in Mueller–Hinton broth.

Bacterial suspensions were adjusted to 0.5 McFarland (1 × 10^8^ CFU/mL). A volume of 180 μL of the suspension was dispensed into each well of a sterile 96-well microtiter plate. The 96-pin lid was then placed onto the plate, ensuring direct contact between each pin and the bacterial suspension. Plates were incubated at 37 °C for 24 h under controlled aerobic conditions. In accordance with the original methodology, polystyrene pin lids (Nunc-Immuno TSP Lids, Thermo Fisher Scientific Inc., Waltham, MA, USA) were used as the pin material. Following incubation, the pin lid was transferred to a fresh 96-well plate containing Mueller–Hinton broth and incubated for an additional 48 h at 37 °C, allowing further maturation of the biofilm on the pin surfaces.

#### 2.5.5. Treatment of Preformed Biofilms with ω-Hydroxylated Phosphonium Ionic Liquids

After biofilm maturation, the pin lid was transferred to a new microplate containing a total volume of 200 μL per well, consisting of 100 μL of each ω-hydroxylated phosphonium ionic liquid and 100 μL of Mueller–Hinton broth, prepared at the same concentrations used in the MIC assays. A fixed concentration above the MIC (0.5 mM) was used to evaluate biofilm disruption, as higher concentrations are generally required to overcome extracellular polymeric substance (EPS)-related barriers and promote matrix destabilization rather than bacterial growth inhibition. Vehicle control wells containing the corresponding DMSO concentration were included to assess any potential effect of the solvent on biofilm formation and disruption. Plates were incubated for 24 h at 37 °C to assess the ability of each compound to disrupt preformed biofilms.

Biofilm biomass was quantified using 0.1% crystal violet (CV) staining [37]. The pin lids were washed three times by immersion in sterile 0.9% saline to remove planktonic or loosely attached cells. The plates were then placed onto a new microplate containing 0.1% crystal violet and incubated for 1 h at 37 °C. After staining, the pins were washed three more times with sterile saline to remove excess dye without disturbing the adhered biofilm.

Crystal violet bound to the biofilm was eluted by transferring the pin lid onto a microplate containing 98% ethanol, followed by incubation for 15 min. The resulting eluate was quantified by measuring absorbance at 595 nm using a microplate reader. The obtained OD595 values were used to calculate the biofilm disruption relative to untreated biofilm controls. Experiments were performed in triplicate, and results are expressed as mean ± standard deviation. Statistical analysis was carried out using one-way ANOVA followed by Tukey’s post hoc multiple-comparison test.

### 2.6. Hemolysis Assay

To assess the cytotoxicity of the compounds, a percent hemolysis assay was performed. Peripheral blood anticoagulated with EDTA was collected from healthy O Rh(+) volunteers [38]. Whole blood was centrifuged at 3000 rpm for 5 min to obtain the erythrocyte pellet. The erythrocytes were washed three times with phosphate-buffered saline (PBS; 10 mM sodium phosphate, 150 mM sodium chloride, pH 7.8 ± 0.2), carefully discarding the supernatant after each wash. Finally, the erythrocytes were resuspended in PBS to obtain a 10% (*v*/*v*) suspension. The compounds were dissolved in PBS to obtain concentrations ranging from 500 to 5 µM. Each dilution was mixed at a 1:1 ratio with the erythrocyte suspension (10% *v*/*v*) in a microplate. The plates were gently shaken and incubated at 37 °C for 1 h. After incubation, the samples were centrifuged at 3000 rpm for 5 min, and the absorbance of the supernatant was measured at 540 nm using a microplate spectrophotometer. The percentage of hemolysis was calculated relative to the complete hemolysis induced by Tween 20 (100% hemolysis, positive control). PBS containing 1% (*w*/*v*) DMSO was used as the negative control. All assays were performed in triplicate at each concentration.

The percentage of hemolysis was determined using the following equation [39]:

Hemolysis %: (*OD_t_* − *OD_nc_*)/(*OD_pc_* − *OD_nc_*) × 100

where *OD_t_* represents the absorbance of the test sample, *OD_pc_* corresponds to the positive control, and *OD_nc_* denotes the negative control.

## 3. Results and Discussion

The different triphenyl and trihexylphosphonium ionic liquids were synthesized according to Scheme 1. The quaternization reactions of phosphines **1** and **4** with the different bromoalkyl alcohols **2a**–**d** (a: *n* = 1; b: *n* = 4; c: *n* = 5; d: *n* = 8) afforded ionic liquids **3a**–**d** and **5a**–**d** in 63–93% yield. The reactions were performed in a sealed vessel and heated at 160 °C for 30 min in a Monowave 50 synthesis reactor (Anton Paar GmbH; Graz, Austria), providing a rapid and efficient route to the desired phosphonium salts.

This reaction is commonly reported to occur over long periods of time, between 24 and 48 h, and at high temperatures under reflux, which implies a high energy cost. Also, the overheating of haloalkanes can promote elimination or reverse quaternization processes, which negatively affect both the yield and purity of the resulting phosphonium salts. These limitations have motivated the development of more efficient synthetic approaches for phosphonium ionic liquids [40,41,42].

The FT-IR spectra of the THP ionic liquid series (Supporting Information Figures S9–S12) confirmed the formation of the phosphonium salts and the presence and integrity of the ω-hydroxyl terminal functionality. All compounds exhibited a broad absorption band in the 3210–3340 cm^−1^ region, assigned to O–H stretching vibrations of the ω-hydroxyl terminal group.

The C3OH derivative showed a more pronounced redshift at 3211 cm^−1^, suggesting stronger hydrogen-bonding interactions and a more polar microenvironment in comparison to the longer-chain analogs (C6–C10), which displayed bands at 3332, 3337, and 3328 cm^−1^, respectively. The strong absorptions at 2950–2850 cm^−1^ were attributed to aliphatic C–H stretching vibrations of methylene (-CH_2_-) and methyl (-CH_3_) groups, with increasing intensity in the order C3 < C6 < C7 < C10 for the THP ionic liquid series. The bands around 1460–1370 cm^−1^ corresponded to CH_2_ bending vibrations, while the intense absorption at 1110–1050 cm^−1^ was assigned to C–O stretching of the primary alcohol group. Additional absorptions in the 1250–1200 cm^−1^ region were associated with P–C stretching of the quaternary phosphonium center.

To verify the influence of the structure on the thermal degradation of the THP and TPP phosphonium ionic liquid series, thermal analysis of the compounds was undertaken in an interval of 50–600 °C in a nitrogen atmosphere.

**Figure 1:**
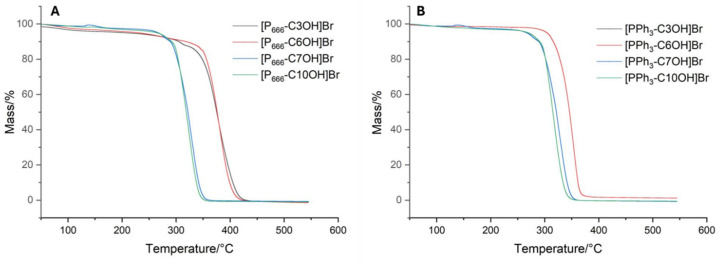
TG curves of the THP (**A**) and TPP (**B**) ionic liquid series.

**Figure 2:**
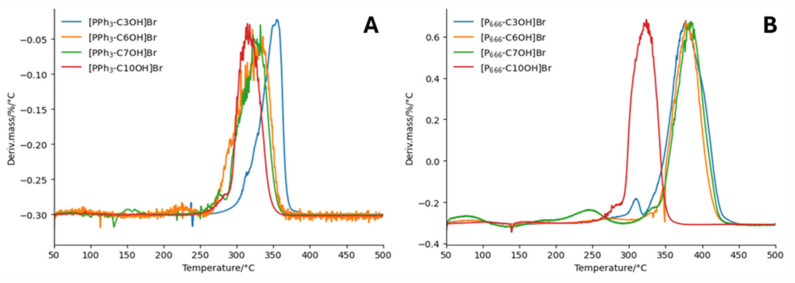
DTG curves of the TPP (**A**) and THP (**B**) ionic liquid series.

The mass loss (TG) and derived mass (DTG) curves of the THP and TPP phosphonium ionic liquid series are presented in Figure 1 and Figure 2, respectively. A plateau is observed in both ionic liquid series, indicating thermal stability up to ~295–327 °C for the TPP and ~298–353 °C for the THP series.

TG analysis of the TPP ionic liquids revealed a minor mass loss of 0.79–2.22% in comparison to 0.85–3.07% mass loss of the THP ionic liquid series, consistent with water loss (Δ*m* ≤ 150 °C). The onset temperature of mass loss (*T_onset_*) is affected by the increase in chain length; thus, the *T_onset_* values of the C3OH and C6OH of the TPP series were 326.76 °C for both, while the *T_onset_* decreased for C10OH < C7OH, with values of 295.93 °C and 297.20 °C, respectively. This behavior was also observed at maximum degradation temperatures (*T_max_*), in which the *T_max_* values of the C3OH and C6OH of the TPP series were 354.67 °C for both, while the *T_max_* decreased for C10OH < C7OH, with values of 314.56 °C and 332.40 °C (Figure 2A), respectively.

On the other hand, the THP ionic liquid series exhibits *T_onset_* and *T_max_* much higher than their TPP counterparts. The *T_onset_* value for C3OH of the THP series (Figure 2B) is 347.14 °C, while the *T_onset_* value decreased in the order C7OH < C6OH, with values of 297.56 °C and 353.49 °C, respectively, while the *T_onset_* for C10OH is the same as that for C7OH (297.56 °C). The *T_max_* values decrease with an increase in chain length, in the order C3OH > C6OH > C7OH > C10OH, with values of 377.48, 376.93, 332.80, and 323.74 °C, respectively.

The decrease in *T_onset_* and *T_max_* observed with the increase in chain length suggests that the hydroxyalkyl moiety present in the TPP and THP ionic liquid series slightly destabilizes the phosphonium framework. This effect is more pronounced in the TPP ionic liquid series. While the enhanced thermal and synthetic stability of phosphonium ionic liquids facilitates handling and reproducibility, it should be distinguished from environmental persistence. Structurally related quaternary cationic antimicrobials have been identified as emerging contaminants due to their stability and persistence, with potential ecological impact and contribution to resistance development [7,9]. Therefore, stability should be considered as a balance between practical applicability and long-term effects. The presence of ω-hydroxylated chains may help modulate this balance, although further studies are required.

Thermal degradation of phosphonium ionic liquids is commonly initiated by reactions occurring on the alkyl substituents, including β-elimination or C–P bond cleavage processes, which can be facilitated by increased chain flexibility or functionalization of the side chain [43,44].

**Figure 3:**
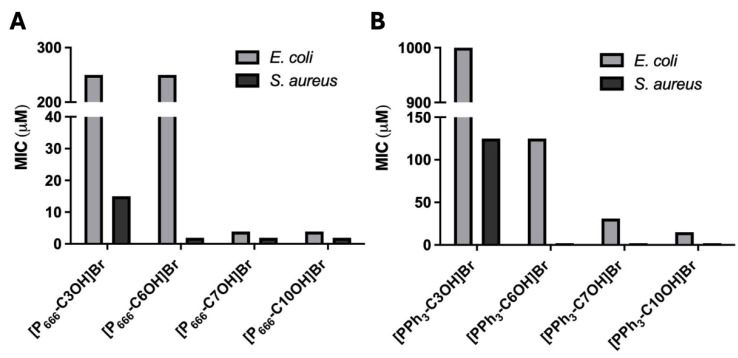
Minimum inhibitory concentration (MIC) of the different THP (**A**) and TPP (**B**) ionic liquid series against *E. coli* and *S. aureus*.

MIC values were expressed in both µM and µg/mL to facilitate direct comparison with previously reported data and clinically relevant reference frameworks. For *S. aureus*, THP and TPP ionic liquids bearing C6OH, C7OH, and C10OH side chains displayed identical MIC values (1.95 µM, Table 1 and Figure 3), suggesting that once a threshold amphiphilicity is reached, further increases in chain length or changes in the phosphonium moiety do not significantly enhance antibacterial potency [45,46].

Apparent lipophilicity values (logD proxy) were estimated using a fragment-based incremental hydrophobicity approach, in which successive alkyl chain elongation was translated into relative hydrophobic contributions within each phosphonium scaffold. Such additive hydrophobic descriptors have been widely used in structure–activity relationship studies as comparative indices rather than as absolute partition coefficients [46,47]. The estimated logD proxy values were [P_666_-C3OH]Br = 2.5, [P_666_-C6OH]Br = 3.85, [P_666_-C7OH]Br = 4.30, [P_666_-C10OH]Br = 5.65, [PPh_3_-C3OH]Br = 2.0, [PPh_3_-C6OH]Br = 3.35, [PPh_3_-C7OH]Br = 3.80, and [PPh_3_-C10OH]Br = 5.15. These values showed the increased hydrophobic contribution resulting from aliphatic chain length extension (C3–C10), as well as scaffold-dependent features, including the flexible aliphatic character of the trihexylphosphonium core and the rigid aromatic surface of the triphenylphosphonium framework. In *S. aureus*, the absence of an outer membrane reduces permeability constraints, and once compounds achieve sufficient amphiphilicity, further increases in hydrophobicity do not appear to proportionally enhance antibacterial activity. Antibacterial effects may become limited by downstream processes such as saturation of membrane binding sites, maximal bilayer perturbation, or rapid attainment of a critical surface concentration [47,48]. This leads to the apparent activity plateau observed across C6OH–C10OH derivatives, regardless of cation core.

Notably, short C3OH derivatives were markedly less active, indicating that below the amphiphilicity threshold (corresponding to lower effective logD values), the molecules may not possess the physicochemical properties required to achieve comparable antibacterial activity [49,50]. The presence of the terminal hydroxyl group further modulates this balance. While it may promote hydrogen bonding at the membrane interface, it simultaneously increases polarity and lowers logD, thereby penalizing lipid partitioning. This effect becomes particularly evident for shorter chains, where the hydrophobic contribution is already limited.

In contrast, *E. coli* exhibited a pronounced dependence on both chain length and cation core. Maximal activity was achieved with C7OH and C10OH derivatives, and trihexylphosphonium analogs were substantially more potent than their triphenyl counterparts (3.9 µM vs. 31–15 µM, Table 1 and Figure 3). This pattern suggests that antibacterial activity may be influenced by outer membrane permeability, potentially involving an interplay between initial electrostatic interactions with the anionic surface and the ability of the compounds to achieve sufficient lipophilicity (logD) to interact more effectively with bacterial cells.

Within this framework, the trihexylphosphonium core provides a more hydrophobic, membrane-compatible cationic scaffold, increasing effective logD (C3OH: 2.5 < C6OH: 3.85 < C7OH: 4.30 < C10OH: 5.65) and enhancing lipid partitioning once surface association occurs. Conversely, the triphenylphosphonium core, although capable of strong localized electrostatic interactions, exhibits comparatively lower effective lipophilicity (C3OH: 2.0 < C6OH: 3.35 < C7OH: 3.80 < C10OH: 5.15) and therefore a reduced capacity to drive the hydrophobic insertion step required to overcome the *E. coli* permeability barrier [51]. The terminal hydroxyl group further decreases logD by increasing polarity, an effect that becomes particularly detrimental in shorter alkyl chains and helps explain the dramatic loss of potency observed for C3OH and C6OH derivatives, especially in the TPP series.

In addition, regression analysis revealed that the TPP ionic liquid series exhibited a clearly stronger linear lipophilicity–activity relationship against *E. coli* (R^2^ = 0.937) compared to the THP ionic liquid series (R^2^ = 0.634) (see Supporting Information Figures S13 and S14). This suggests that antibacterial activity within the TPP scaffold is more predictably governed by hydrophobic partitioning. The aromatic triphenylphosphonium core provides a rigid hydrophobic surface that yields a consistent structure–activity trend, whereas the aliphatic THP core displays a more threshold-driven response.

Collectively, these findings suggest that bacterial envelope-related factors may contribute to the observed activity against *E. coli*, although dedicated mechanistic studies are required to confirm this hypothesis. While THP derivatives reach superior maximal potency at longer chain lengths, TPP analogs are consistent with a more tunable and more linear lipophilicity–activity relationship.

For *S. aureus*, both series showed comparable and moderate correlations (R^2^ = 0.653 for THP and R^2^ = 0.653 for TPP), in agreement with the plateau behavior observed for C6OH–C10OH derivatives (see Supporting Information Figure S14). This reduced dependence on lipophilicity suggests that, in *S. aureus*, once sufficient membrane partitioning is achieved, antibacterial activity becomes less sensitive to further hydrophobic increases within this compound series. Although the broader literature on cationic amphiphiles often reports a strong dependence on alkyl chain length up to an optimal range, the plateau observed here likely reflects a threshold amphiphilicity specific to the studied derivatives. The lack of an outer membrane in *S. aureus* decreases permeability barriers and shifts the limiting step toward membrane perturbation efficiency rather than membrane penetration [52]. Under these conditions, once a critical surface concentration is reached, additional lipophilic contribution does not proportionally enhance bactericidal performance.

MIC values are reported as exact numerical values and expressed in both µM and µg/mL. Vehicle controls containing 0.5% (*v*/*v*) DMSO showed no effect on bacterial growth. The MIC was defined as the lowest concentration showing no visible bacterial growth, confirmed by the absence of TTC reduction.

At 0.5 mM, biofilm disruption assessed by crystal violet staining revealed a dependence on both phosphonium core structure and alkyl chain length, consistent with an amphiphilicity-dependent biofilm disruption profile. Although *S. aureus* showed higher susceptibility under planktonic conditions (lower MIC values), a greater degree of biofilm removal was observed for *E. coli*. This apparent discrepancy can be explained by the different nature of the assays employed. While MIC reflects bacterial growth inhibition, the crystal violet assay quantifies total biofilm biomass (cells and extracellular polymeric substances) without distinguishing viability. Therefore, the higher biofilm removal in *E. coli* likely reflects enhanced disruption or detachment of the biofilm matrix rather than increased antibacterial activity. THP series consistently exhibited superior biomass removal compared with TPP series, particularly against *E. coli* biofilms, where THP derivatives achieved 60–92% disruption versus < 50% for all TPP compounds (Table 2). Because crystal violet quantifies total attached biomass (cells plus EPS), these results suggest that the more hydrophobic THP scaffold likely confers enhanced interfacial activity and higher apparent lipophilicity (logD), favoring partitioning into the EPS matrix of the biofilm. Such behavior may contribute to biofilm matrix destabilization, promoting structural loosening and facilitating biofilm detachment [28,53,54]. A clear chain-length “window” was observed, with [P_666_-C6OH]Br and [P_666_-C7OH]Br derivatives displaying the highest disruption in both species, whereas shorter ([P_666_-C3OH]Br) and longer ([P_666_-C10OH]Br) chains were less effective, particularly in *S. aureus*. This non-monotonic behavior is consistent with an optimal logD range: insufficient hydrophobicity (e.g., C3OH), further attenuated by a terminal hydroxyl group, likely reduces effective lipophilicity and limits penetration into the EPS matrix. Conversely, excessive hydrophobicity (e.g., C10OH) may favor self-aggregation or strong surface adsorption, thereby reducing overall biofilm disruption efficiency and ultimately reducing overall biomass removal [47,49,51]. The lower performance of TPP derivatives across all chain lengths suggests that, although the cationic phosphonium center promotes electrostatic association with negatively charged matrix components, the triphenyl moiety provides less effective hydrophobic insertion and interfacial disruption than the trihexyl analog. Overall, the data suggest that biofilm disruption is associated with physicochemical properties related to amphiphilicity and phosphonium scaffold structure, with THP derivatives bearing C6OH–C7OH chains achieving the most favorable amphiphilic profile for efficient biofilm removal in both *E. coli* and *S. aureus* systems. At the tested concentration (0.5 mM), the observed biofilm biomass disruption may be influenced by non-specific membrane interactions, as suggested by the hemolytic activity at similar concentrations. Therefore, these results should be interpreted as an initial screening of biofilm-disrupting potential rather than an indication of selective anti-biofilm efficacy.

**Figure 4:**
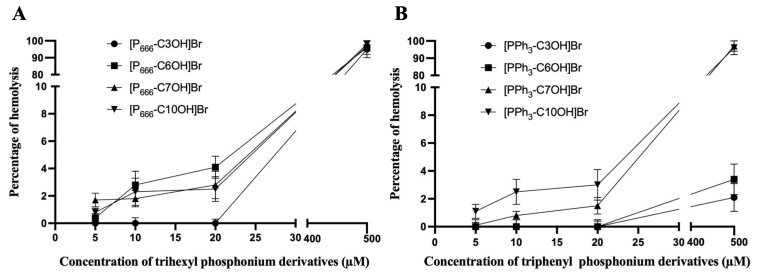
Hemolytic activity of THP (**A**) and TPP (**B**) ionic liquid series.

The hemolytic activity was evaluated as an indicator of potential membrane-related cytotoxicity, given that both antibacterial action and erythrocyte lysis are commonly associated with membrane perturbation mechanisms. At concentrations up to 20 µM—close to or above the MIC values of the most active derivatives—none of the studied ionic liquids exhibited significant hemolytic activity (Figure 4), supporting the existence of a favorable selectivity window toward bacterial membranes. A clear concentration-dependent effect emerged at higher doses, particularly for the more hydrophobic derivatives. In the trihexylphosphonium (THP) series, hemolysis increased progressively with concentration and became pronounced for the C10OH analog at ≥400–500 µM, reaching near-complete erythrocyte lysis. This behavior is consistent with excessive lipophilicity (higher apparent logD) promoting deep insertion into the erythrocyte lipid bilayer and surfactant-like membrane solubilization once a critical concentration is exceeded [50,51]. In contrast, derivatives bearing shorter alkyl chains exhibited markedly lower hemolytic effects, suggesting that insufficient hydrophobicity limits disruptive insertion into mammalian membranes. The triphenylphosphonium (TPP) derivatives displayed consistently lower hemolytic activity than their trihexylphosphonium (THP) counterparts, with [PPh_3_–C3OH]Br and [PPh_3_–C6OH]Br inducing minimal erythrocyte lysis (2.1% and 3.4%, respectively) even at 500 µM. This reduced membrane toxicity likely arises from their lower effective lipophilicity compared to the more hydrophobic THP analogs, limiting deep bilayer insertion. Additionally, stronger ion pairing between the phosphonium cation and its counterion (Br^−^) may reduce the fraction of free cationic species in solution, thereby limiting their effective interaction with biological membranes [28,55]. The presence of the terminal hydroxyl group may further attenuate hemolysis by increasing polarity and lowering apparent logD, thereby limiting deep bilayer partitioning in eukaryotic membranes. Overall, these findings may indicate that hemolytic activity is primarily governed by global amphiphilicity and chain length rather than charge alone, and that derivatives within the C6OH–C7OH range—particularly in the THP series—maintain potent antibacterial activity while exhibiting minimal erythrocyte toxicity at therapeutically relevant concentrations. Although a formal selectivity index (SI) could not be determined due to the absence of half-maximal hemolytic concentration (HC_50_) values, a qualitative assessment indicates a favorable selectivity window. The most active compounds exhibited MIC values in the low micromolar range, whereas significant hemolytic activity was observed only at much higher concentrations, suggesting a high degree of selectivity toward bacterial membranes.

## 4. Conclusions

In this study, two series of hydroxylated triphenylphosphonium (TPP) and trihexylphosphonium (THP) ionic liquids with varying alkyl chain lengths were synthesized and evaluated for their thermal stability and antibacterial and anti-biofilm activities.

The modified synthetic approach reduced reaction times from 24–48 h to 30 min while maintaining high yields (63–93%), demonstrating improved efficiency. Additionally, the thermal analysis revealed that the THP series exhibited superior thermal stability compared to their TPP counterparts, with onset decomposition temperatures in the range of 298–353 °C versus 295–327 °C, respectively. Notably, both series maintained structural integrity up to well above typical biological assay temperatures, while longer hydroxyalkyl chains (C7OH and C10OH) slightly reduced thermal stability in both scaffolds, likely due to increased chain flexibility facilitating β-elimination pathways. These findings support the practical utility of these ionic liquids under standard experimental conditions and highlight THP derivatives as particularly robust candidates for antimicrobial applications.

Indeed, data suggest that antibacterial potency is strongly influenced by the amphiphilic balance of the molecules, particularly by the nature of the phosphonium core and the length of the alkyl side chain. Derivatives bearing C6OH–C10OH chains showed the highest antibacterial activity, while short-chain analogs displayed markedly reduced potency, highlighting the importance of sufficient lipophilicity for effective membrane interaction. Notably, THP derivatives exhibited higher antibacterial activity against *E. coli* compared with their TPP counterparts, consistent with their greater hydrophobicity and enhanced capacity for membrane partitioning.

The ω-hydroxylated phosphonium ionic liquids also demonstrated the ability to disrupt preformed biofilms, with THP derivatives showing the most pronounced biomass removal. The highest anti-biofilm activity was observed for derivatives containing C6OH–C7OH chains, suggesting the existence of an optimal amphiphilic window that favors penetration into the biofilm matrix and promotes biofilm detachment. Importantly, the most active ω-hydroxylated phosphonium ionic liquids exhibited minimal hemolytic activity at concentrations close to their antibacterial MIC values, indicating a favorable selectivity toward bacterial membranes.

Overall, these findings highlight ω-hydroxylated phosphonium ionic liquids as promising antimicrobial agents with likely membrane-related activity and provide valuable structure–activity insights for the rational design of new antibacterial and anti-biofilm compounds. While their thermal and synthetic stability support practical applicability, future studies should address their environmental fate and potential contribution to resistance development to ensure safe and sustainable use.

## Figures and Tables

**Table 1:** Minimum inhibitory concentration (MIC) values (µg/mL and µM) of hydroxylated phosphonium ionic liquids against *E. coli* and *S. aureus*.

	*E. coli*		*S. aureus*	
	µg/mL	µM	µg/mL	µM
[P_666_-C3OH]Br	106.37	250.0	6.38	15.0
[P_666_-C6OH]Br	116.89	250.0	0.91	1.95
[P_666_-C7OH]Br	1.88	3.9	0.94	1.95
[P_666_-C10OH]Br	2.04	3.9	1.02	1.95
[PPh_3_-C3OH]Br	401.28	1000.0	50.16	125.0
[PPh_3_-C6OH]Br	55.42	125.0	0.86	1.95
[PPh_3_-C7OH]Br	14.18	31.0	0.89	1.95
[PPh_3_-C10OH]Br	7.49	15.0	0.97	1.95

**Table 2:** Biofilm disruption activity (%) of the different THP and TPP ionic liquid series at 0.5 mM against *E. coli* and *S. aureus* preformed biofilms.

	*E. coli*	*S. aureus*
Molecule 0.5 mM	% of Disruption	
[P_666_-C3OH]Br	80.5% ± 2.1% ^a^	0.2% ± 0.1% ^a^
[P_666_-C6OH]Br	91.7% ± 1.7% ^b^	68.8% ± 3.5% ^b^
[P_666_-C7OH]Br	88.8% ± 3.6% ^b^	64.9% ± 2.6% ^b^
[P_666_-C10OH]Br	59.7% ± 2.6% ^c^	22.3% ± 1.8% ^c^
[PPh_3_-C3OH]Br	44.9% ± 1.4% ^A^	0.1% ± 0.1% ^A^
[PPh_3_-C6OH]Br	47.8% ± 3.4% ^A^	33.1% ± 2.1% ^B^
[PPh_3_-C7OH]Br	48.6% ± 3.1% ^A^	41.0% ± 3.4% ^C^
[PPh_3_-C10OH]Br	17.3% ± 2.2% ^B^	20.8% ± 2.2% ^D^

Data are expressed as mean ± SD. Letters indicate statistical groups based on adjusted *p*-values from one-way ANOVA followed by Tukey’s post hoc test. Values sharing at least one letter are not significantly different; values with no letters in common are significantly different (*p* ≤ 0.05). Lowercase letters compare compounds within the THP series; uppercase letters compare compounds within the TPP series. Cross-series comparisons were not assigned because they were not included in the provided Tukey output.

## Data Availability

The original contributions presented in this study are included in the article. Further inquiries can be directed to the corresponding author.

## Supplementary Materials

The following supporting information can be downloaded at: https://www.mdpi.com/article/10.3390/antibiotics15070655/s1.
