# Supplementary material for: Hydroxylated Alkyl and Phenyl Phosphonium Ionic Liquids Exhibit Enhanced Antibacterial and Anti-Biofilm Activity
Source: Antibiotics (Basel). 2026 Jul 1;15(7):655. doi: 10.3390/antibiotics15070655 (PMC13405887; doi:10.3390/antibiotics15070655)
Supplement: Supplementary file 1 [file antibiotics-15-00655-s001.zip › antibiotics-4246433-supplementary.pdf]

Supporting Information for

Hydroxylated Alkyl and Phenyl Phosphonium Ionic Liquids Exhibit Enhanced Antibacterial and Anti-Biofilm Activity

Oscar Forero-Doria[1], Rosío Rodríguez-Azúa[2,3], Maria Parot-Cabrera[1,4], Verónica Olate-Olave[1], Christina Mitsi[5], Ricardo I. Castro[6], Matías Monroy-Cárdenas[7], Whitney Venturini[8], Ramiro Araya-Maturana[9,10], Luis Guzmán[1*]

[1]Departamento de Bioquímica Clínica e Inmunohematología, Facultad de Ciencias de la Salud, Universidad de Talca, Talca 3460000, Chile; oforero@utalca.cl (O.F.-D.); javiparot@gmail.com (M.P.-C.); volate@utalca.cl (V.O.-O.)

[2]Oral and Maxillofacial Histopathology Laboratory, Department of Stomatology, Faculty of Dentistry, Universidad de Talca, Talca 3460000, Chile; rorodriguez@utalca.cl

[3]Doctorado en Ciencias Biomédicas, Facultad de Ciencias de la Salud, Universidad de Talca, Talca 3460000, Chile

[4]Magister en Ciencias Biomédicas, Facultad de Ciencias de la Salud, Universidad de Talca, Talca 3460000, Chile

[5]Center for Bioinformatics and Integrative Biology, Facultad de Ciencias de la Vida, Universidad Andrés Bello, Santiago 8370146, Chile; christina.mitsi@usach.cl

[6]Multidisciplinary Agroindustry Research Laboratory, Carrera de Ingeniería en Construcción, Instituto de Ciencias Aplicadas, Universidad Autónoma de Chile, Talca 3467987, Chile; ricardo.castro@uautonoma.cl

[7]Laboratorio de Síntesis y Reactividad de Compuestos Orgánicos, Departamento de Química, Facultad de Ciencias Exactas, Universidad Andrés Bello, Santiago 8370146, Chile; maty.monroy@gmail.com

[8]Departamento de Medicina Traslacional, Facultad de Medicina, Universidad Católica del Maule, Talca 3460000, Chile; wventurini@ucm.cl

[9]MIBI (Interdisciplinary Group on Mitochondrial Targeting and Bioenergetics), Universidad de Talca, Talca 3460000, Chile; raraya@utalca.cl

[10]Instituto de Química de Recursos Naturales, Universidad de Talca, Talca 3460000, Chile

*Correspondence:

Luis Guzmán

lguzman@utalca.cl

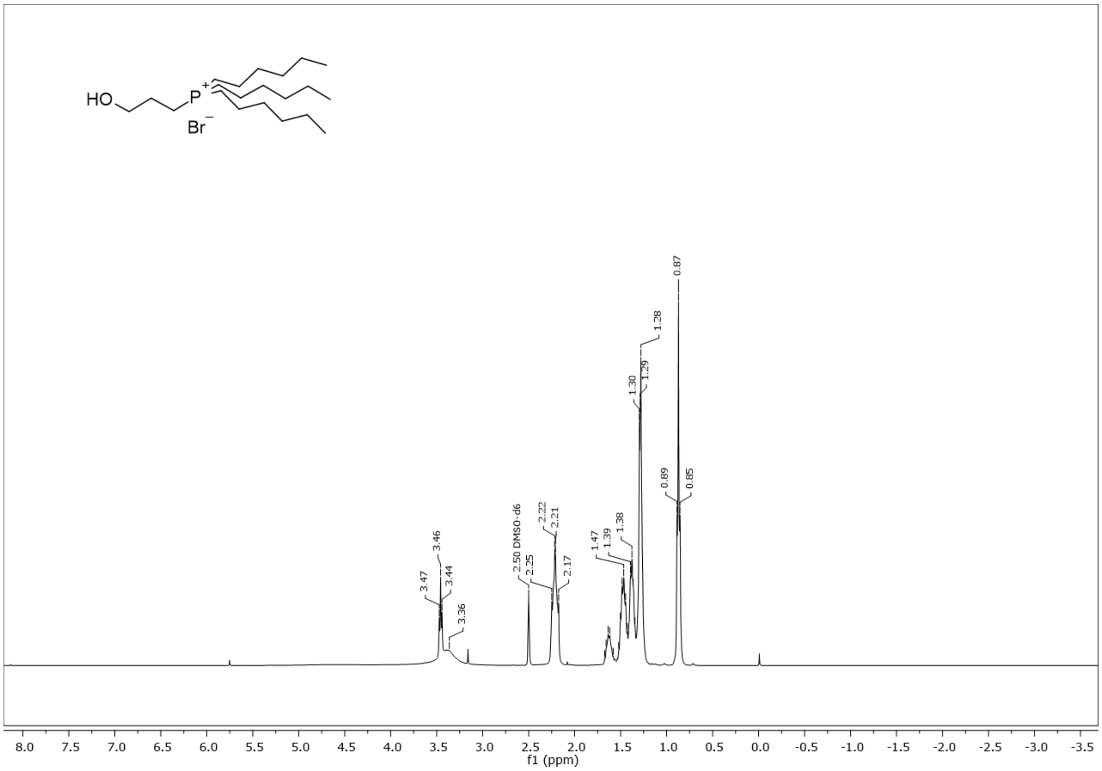

Fig.S1. ^{1}H-NMR spectrum of (3-Hydroxypropyl)trihexylphosphonium bromide (**5a**)

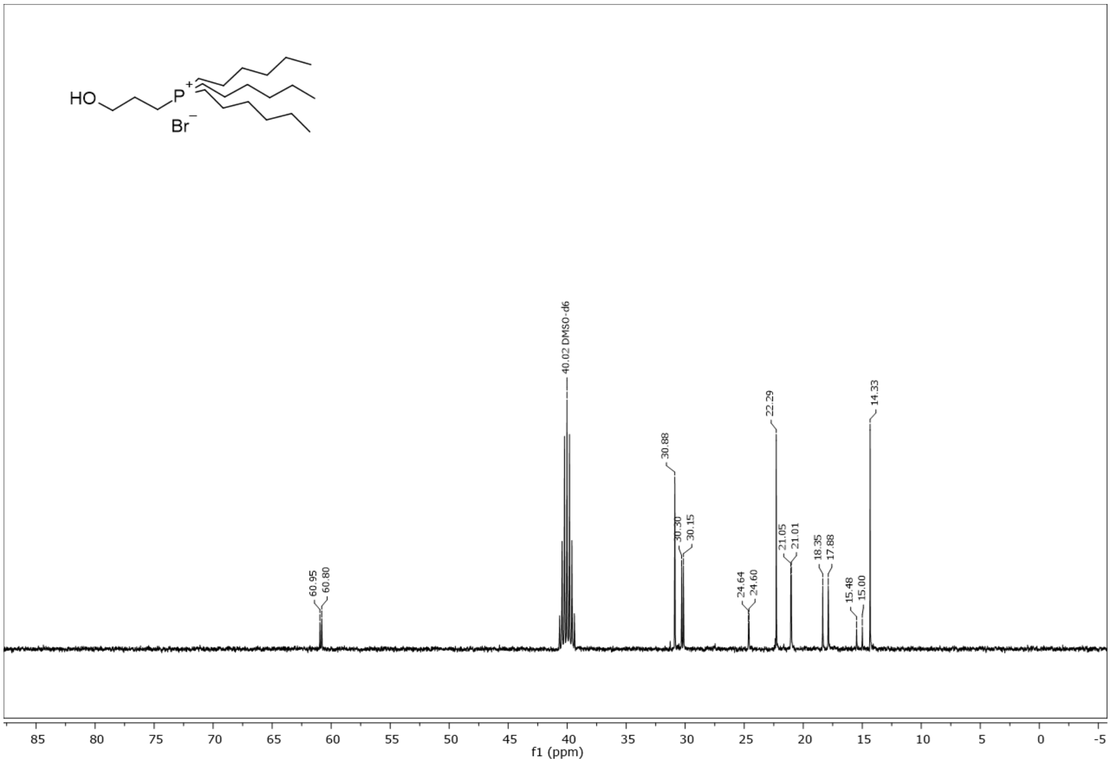

Fig.S2. ^{13}C-NMR spectrum of (3-Hydroxypropyl)trihexylphosphonium bromide (**5a**)

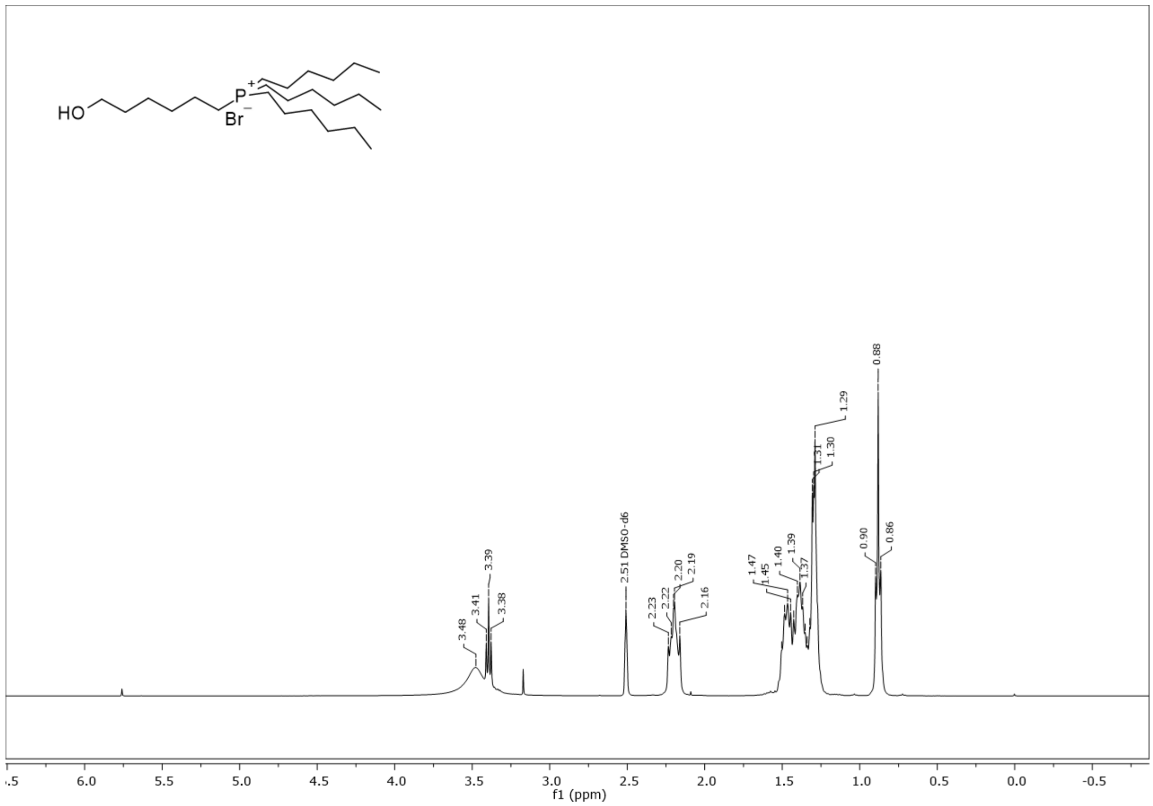

Fig.S3. ^{1}H-NMR spectrum of (6-Hydroxypropyl)trihexylphosphonium bromide (**5b**)

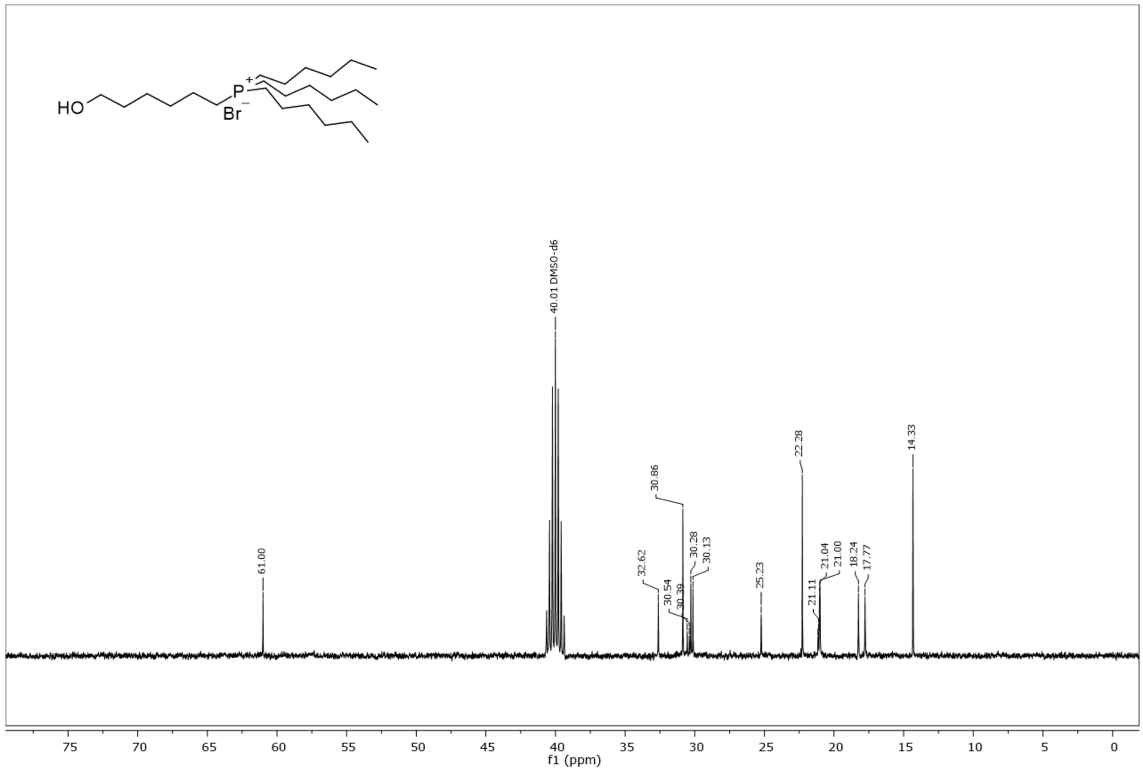

Fig.S4. ^{13}C-NMR spectrum of (6-Hydroxypropyl)trihexylphosphonium bromide (**5b**)

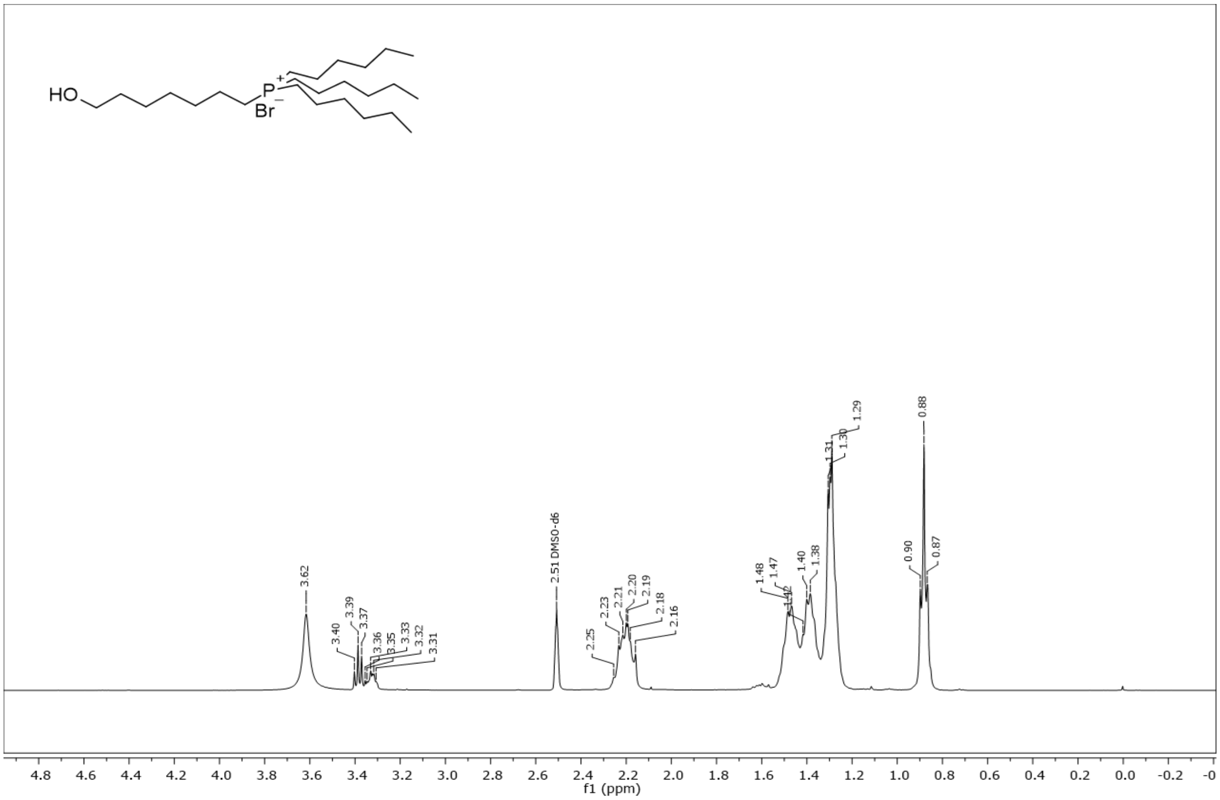

Fig.S5. ^{1}H-NMR spectrum of (7-Hydroxypropyl)trihexylphosphonium bromide (**5c**)

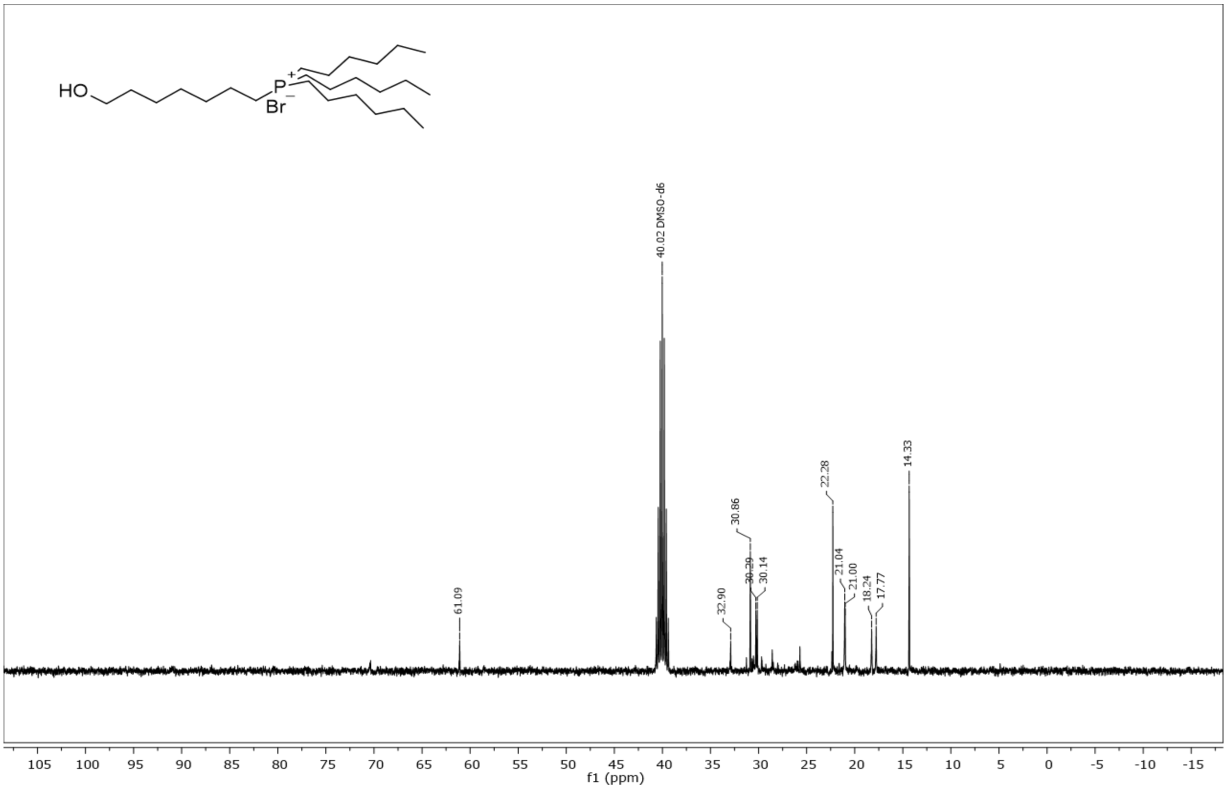

Fig.S6. ^{13}C-NMR spectrum of (7-Hydroxypropyl)trihexylphosphonium bromide (**5c**)

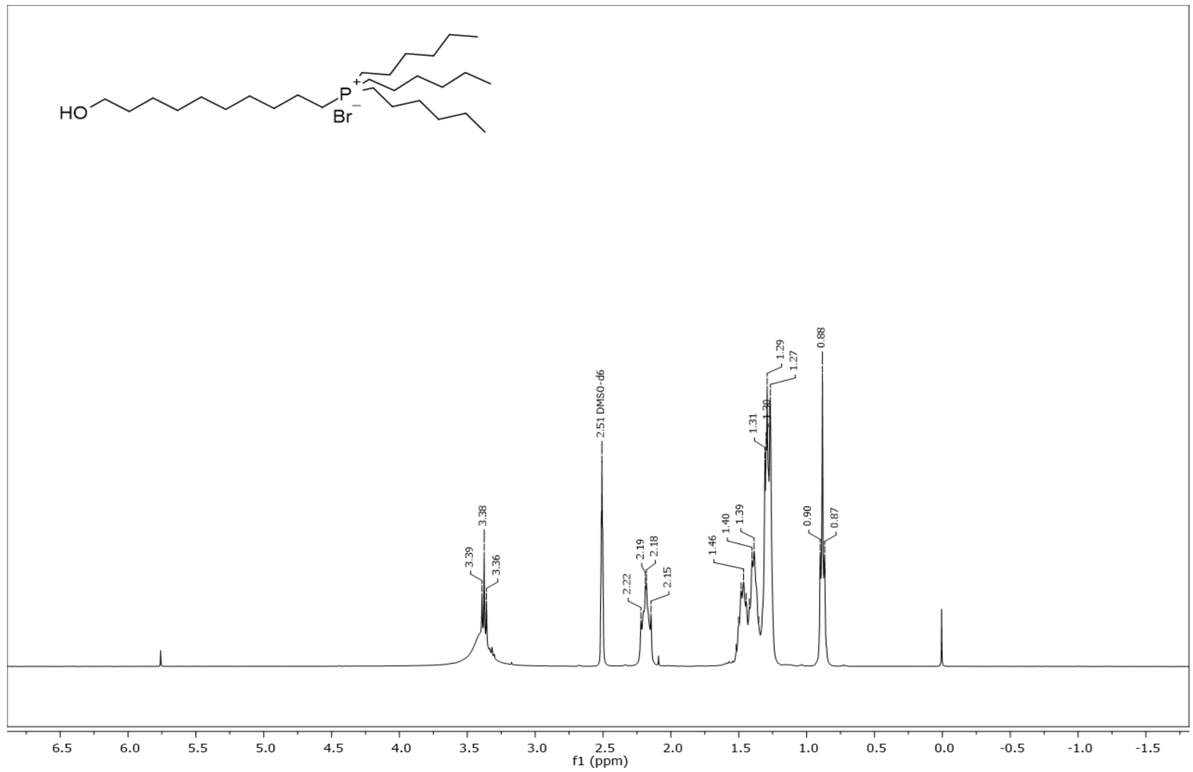

Fig.S7. ^{1}H-NMR spectrum of (10-Hydroxypropyl)trihexylphosphonium bromide (**5d**)

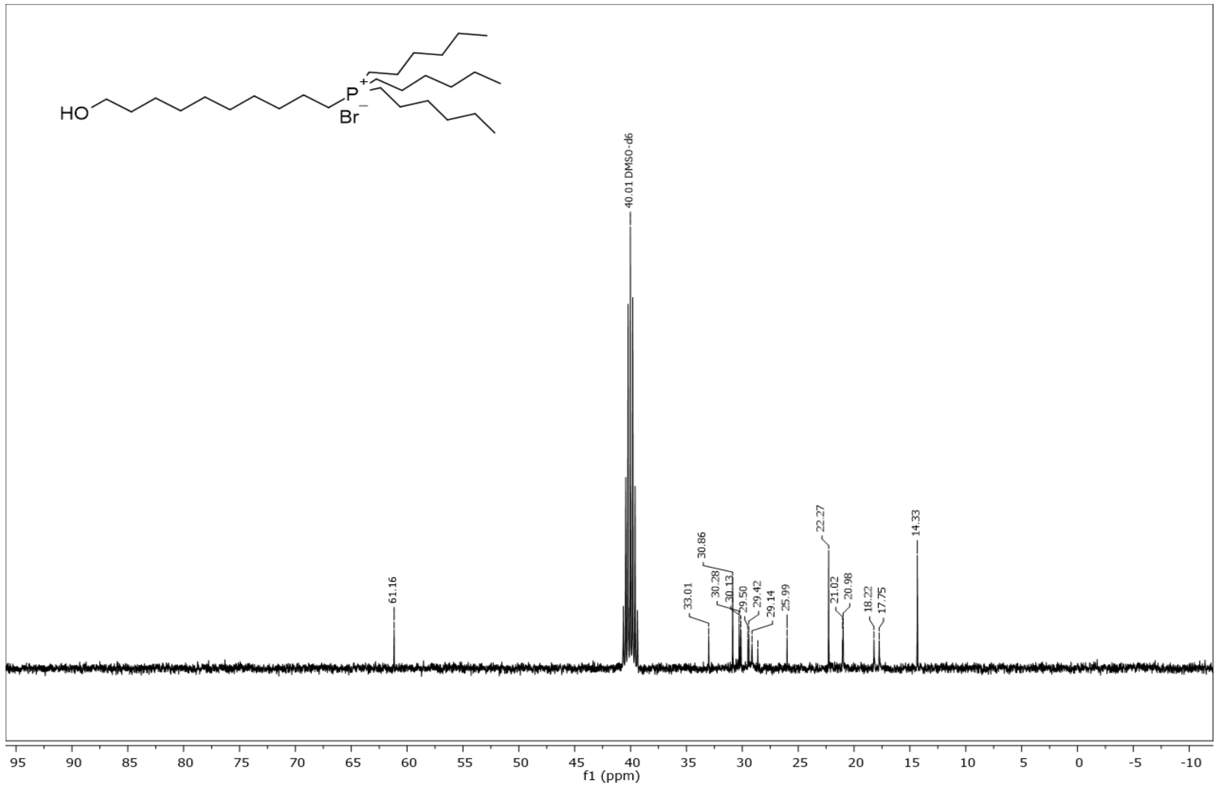

Fig.S8. ^{13}C-NMR spectrum of (10-Hydroxypropyl)trihexylphosphonium bromide (**5d**)

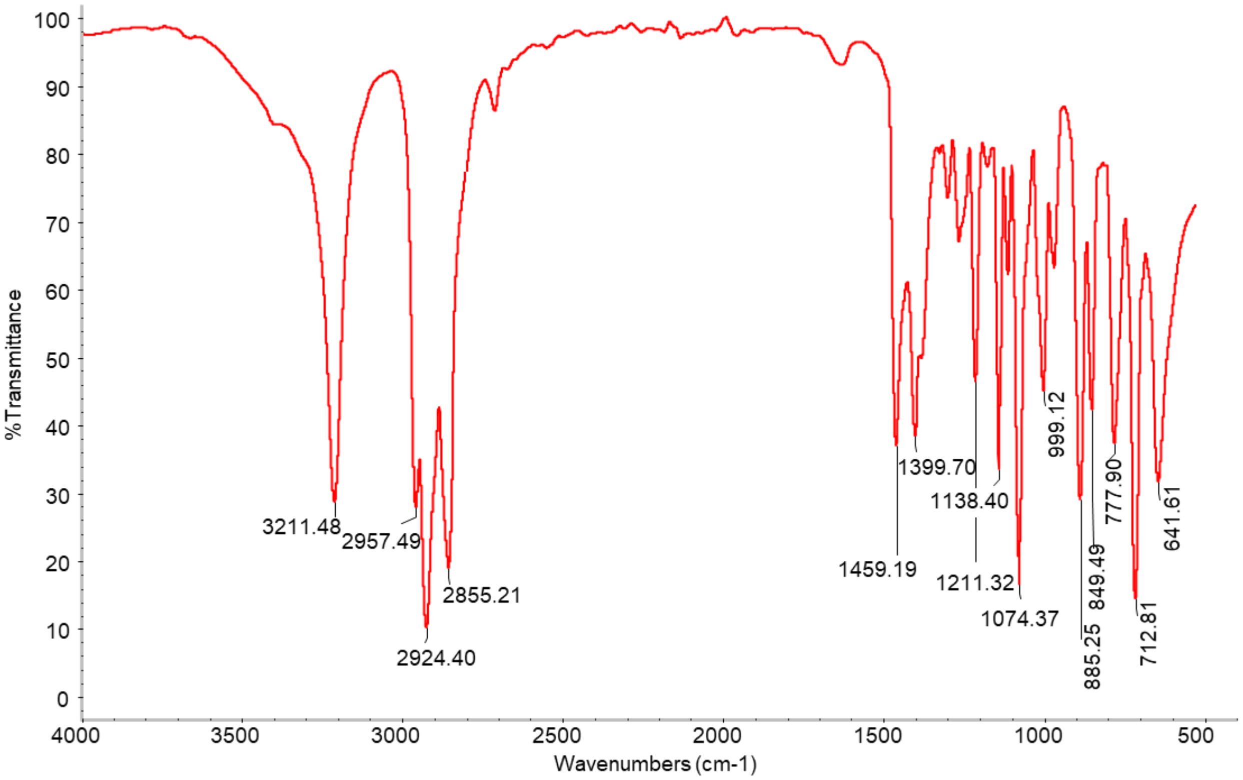

Fig.S9. ATR-FTIR spectrum of (3-Hydroxypropyl)trihexylphosphonium bromide (**5a**)

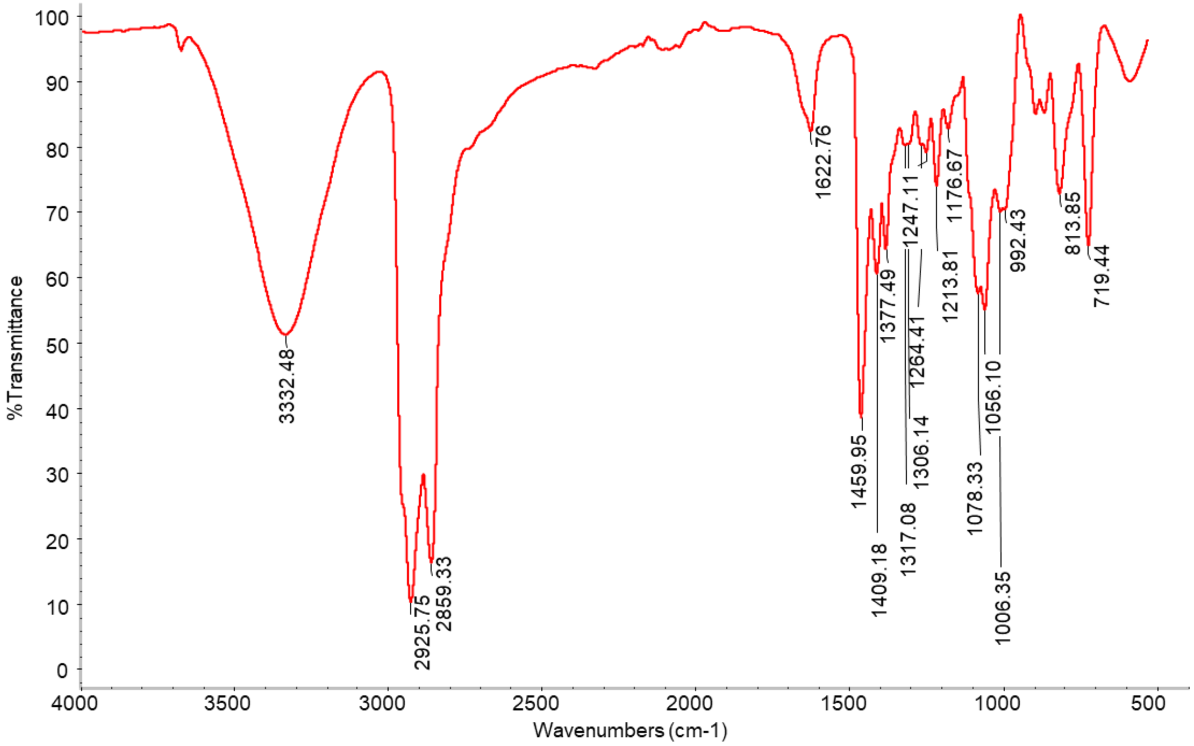

Fig.S10. ATR-FTIR spectrum of (6-Hydroxypropyl)trihexylphosphonium bromide (**5b**)

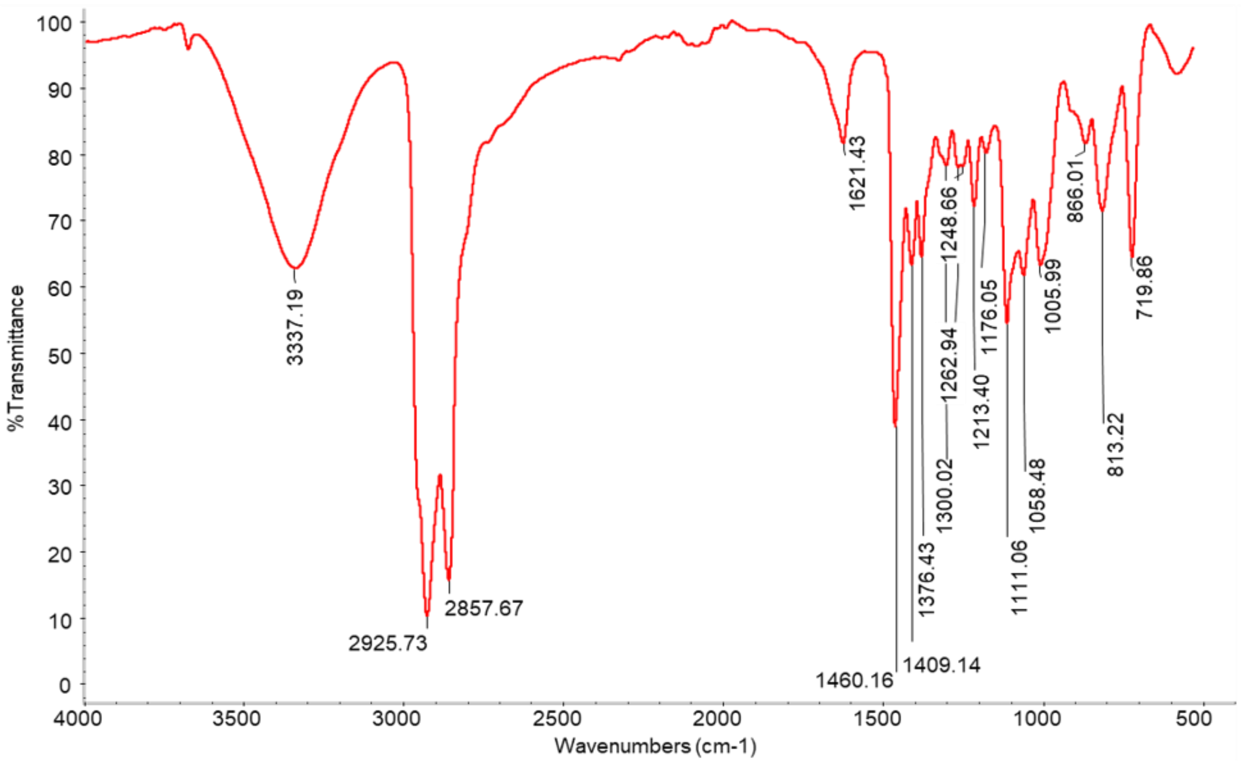

Fig.S11. ATR-FTIR spectrum of (7-Hydroxypropyl)trihexylphosphonium bromide (**5c**)

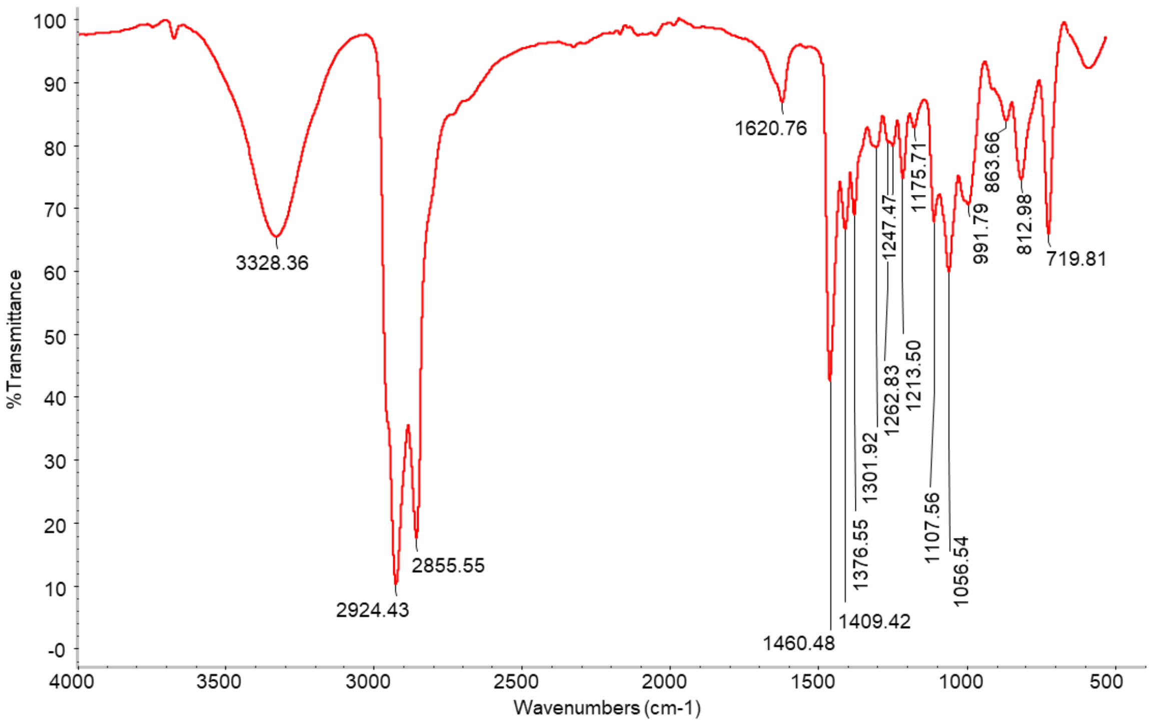

Fig.S12. ATR-FTIR spectrum of (10-Hydroxypropyl)trihexylphosphonium bromide (**5d**)

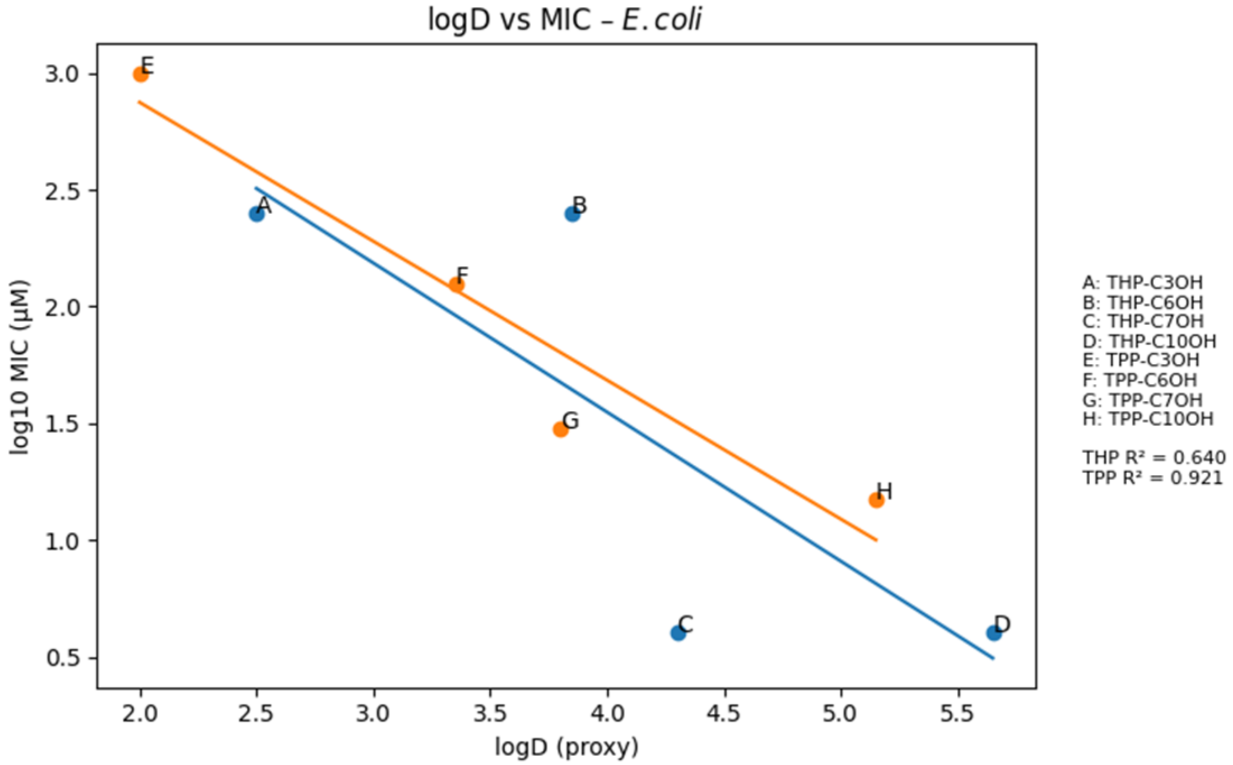

Fig.S13. Relationship between lipophilicity and antibacterial activity against *E. coli* for TPP and THP ionic liquid series. A stronger linear correlation was observed for TPP ($R^2 = 0.937$) than for THP ($R^2 = 0.634$).

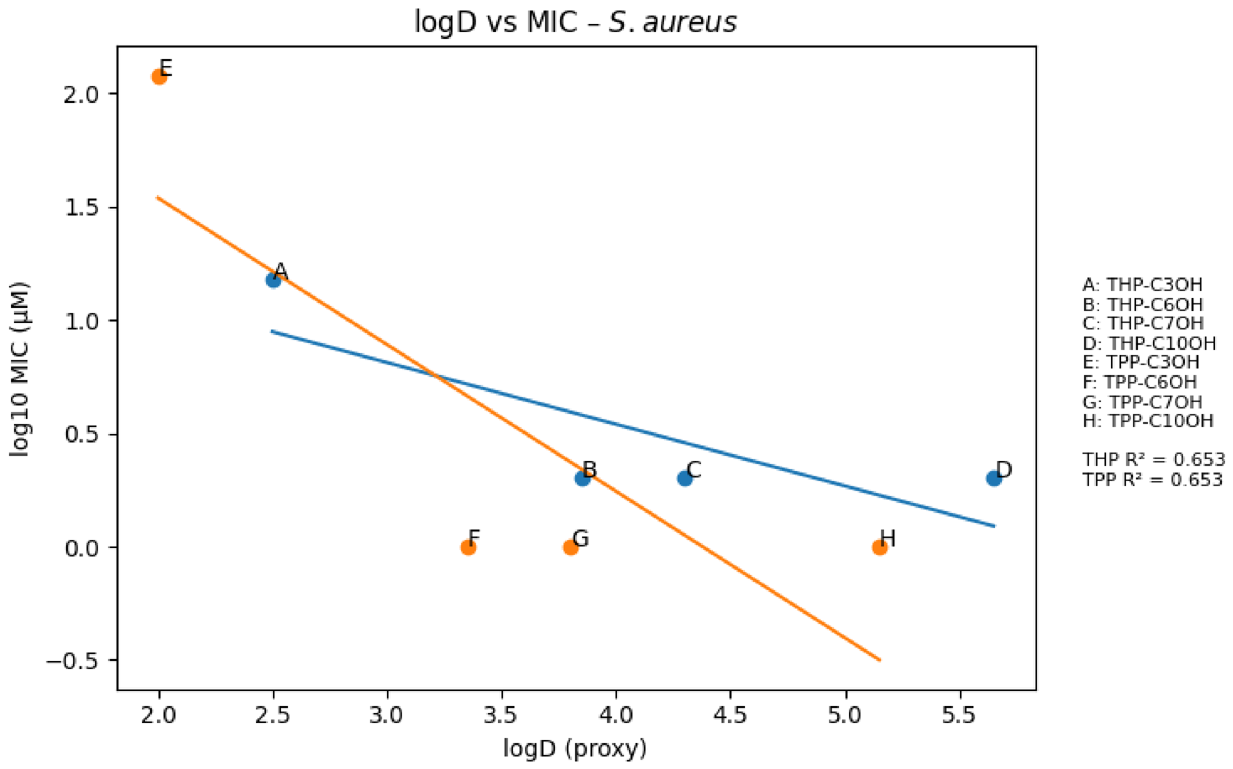

Fig.S14. Relationship between lipophilicity and antibacterial activity against *S. aureus* for TPP and THP ionic liquid series. Both series showed similar moderate correlations ($R^2 = 0.653$).
